# Insights into the Molecular Pathogenesis of Activated B-Cell-like Diffuse Large B-Cell Lymphoma and Its Therapeutic Implications

**DOI:** 10.3390/cancers7020812

**Published:** 2015-05-22

**Authors:** Georg Lenz

**Affiliations:** 1 Translational Oncology, Department of Medicine A, Albert-Schweitzer Campus 1, University Hospital Münster, 48149 Münster, Germany; E-Mail: georg.lenz@ukmuenster.de; Tel.: +49-251-83-52995; Fax: +49-251-83-52673; 2 Cluster of Excellence EXC 1003, Cells in Motion, 48149 Münster, Germany

**Keywords:** ABC DLBCL, NF-κB pathway, B-cell receptor signaling, molecular targets

## Abstract

Within the last couple of years, the understanding of the molecular mechanisms that drive the pathogenesis of diffuse large B-cell lymphoma (DLBCL) has significantly improved. Large-scale gene expression profiling studies have led to the discovery of several molecularly defined subtypes that are characterized by specific oncogene addictions and significant differences in their outcome. Next generation sequencing efforts combined with RNA interference screens frequently identify crucial oncogenes that lead to constitutive activation of various signaling pathways that drive lymphomagenesis. This review summarizes our current understanding of the molecular pathogenesis of the activated B-cell-like (ABC) DLBCL subtype that is characterized by poor prognosis. A special emphasis is put on findings that might impact therapeutic strategies of affected patients.

## 1. Introduction

Diffuse large B-cell lymphoma (DLBCL) is the most common malignant lymphoma subtype in adults, accounting for roughly 40% of all cases [1]. DLBCL is characterized by remarkable heterogeneity, affecting several aspects of the disease. Morphologically diverse variants can be distinguished. The distinction of these variants potentially mirrors differences in biology and might also be clinically relevant, as for example the immunoblastic variant is associated with adverse outcome [2]. Besides these pathological differences, patients are also characterized by variable clinical presentation. Finally, there is heterogeneity with respect to the molecular events that drive DLBCL lymphomagenesis. This heterogeneity can at least partially be explained by the existence of molecularly defined subtypes identified in large gene expression profiling studies in recent years [3,4,5,6,7,8]. By applying the cell of origin classification, in which the DLBCL tumor profiles are compared to profiles of normal B-cells, several molecular subtypes can be distinguished. The germinal center B-cell-like (GCB) DLBCLs are derived from germinal center B-cells and, accordingly, they express a variety of genes that are expressed in normal germinal center B-cells. In contrast, activated B-cell-like (ABC) DLBCLs seem to originate from activated B-cells that are in the transition of being differentiated into plasma cells [3,6,9]. Primary mediastinal B-cell lymphomas (PMBLs) seem to originate from a B-cell subpopulation in the thymus and are characterized by the virtue of a specific gene expression profile [4,5,10]. Finally, approximately 15% of DLBCLs cannot be assigned to a molecular subtype and are referred to as unclassifiable DLBCL [11]. Especially, the distinction of ABC and GCB DLBCLs is not only relevant from a scientific standpoint, but additionally also has significant clinical implications, as these subtypes are characterized by differences in overall survival when treated with the current standard treatment of rituximab and CHOP chemotherapy (R-CHOP) [11]. The vast majority of patients diagnosed with GCB DLBCL respond favorably to R-CHOP, whereas R-CHOP is less effective in ABC DLBCL patients [11]. These data indicate that a better understanding of the biology of the subtypes is warranted to exploit the molecular vulnerabilities of these lymphomas. Due to the less favorable outcome of ABC DLBCL patients, this review focuses on recent insights into the molecular pathogenesis of ABC DLBCL.

## 2. Insights into the Biology of ABC DLBCL

Various studies of hundreds of tumor specimens derived from ABC DLBCL patients suggested that ABC DLBCL lymphoma cells are in the process of being differentiated into plasma cells [3,6,9]. Inactivation of BLIMP1, the master regulator of plasma cell differentiation, seems to be one of the driving events of this lymphoma subtype [12,13]. BLIMP1 mediates plasma cell differentiation by terminating the expression of a variety of B-cell differentiation genes such as *Spi-B*, *PAX5*, and *Oct-2* [14]. Different molecular mechanisms have been identified that inactivate BLIMP1 [15]. Roughly 25% of ABC DLBCL patient samples are characterized by inactivating mutations of *PRDM1* that encodes BLIMP1 [12,13]. *PRDM1*/BLIMP1 is furthermore deregulated by chromosomal translocations affecting *BCL6* that are found in approximately 25% of patient samples. BCL6 functions as a repressor of *PRDM1* [16]. Finally more than 25% of ABC DLBCL samples show gains/amplifications and overexpression of the transcription factor Spi-B that can also repress BLIMP1 expression [17,18]. The importance of BLIMP1 for the pathogenesis of ABC DLBCL was further underscored by different mouse models that showed that conditional knockout of *BLIMP1* in mouse B-cells promotes the development of lymphoproliferative disorders that have features of human ABC DLBCL [15,19]. Collectively, these data implicate that a block in differentiation is an important event in the molecular pathogenesis of ABC DLBCL.

**Figure 1:**
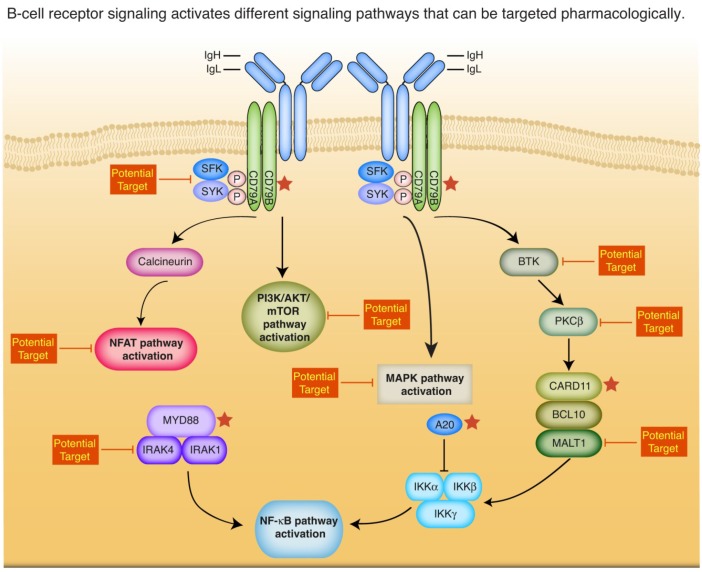
B-cell receptor signaling activates different signaling pathways that can be targeted pharmacologically. Abbreviations: IgH: immunoglobulin heavy chain; IgL: immunoglobulin light chain. Asterisks indicate genes that are targeted by somatically acquired mutations.

Another characteristic hallmark of ABC DLBCL biology is the constitutive activation of the oncogenic nuclear factor-kappa B (NF-κB) signaling pathway (Figure 1) [20]. The NF-κB transcription factor family consists of five different members termed RelA (p65), RelB, c-Rel and p50/p52 with its precursors p105/p100 [21,22,23]. In resting cells, the different NF-κB subunits are normally associated with inhibitory proteins of the IκB family (IκBα, IκBβ, IκBε) or are kept inactive by the precursors p100 and p105 [21]. Upon stimulation, the NF-κB members are released from their inhibitors and subsequently translocate to the nucleus where they activate their target genes [24,25]. Recent work from various scientific groups unraveled that the NF-κB pathway is frequently activated in ABC DLBCL by either gain- or loss-of-function mutations affecting upstream members of the NF-κB signaling cascade [26,27,28,29,30]. Mutations in the ITAM (immunoreceptor tyrosine-based activation motif) motif of the signaling molecule, *CD79B,* that are detectable in approximately 20% of ABC DLBCL cases, or less frequently, deletions affecting the ITAM motifs of *CD79A* are associated with chronic active B-cell receptor (BCR) signaling [27]. Chronic active BCR signaling should be differentiated from tonic BCR signaling. Antigen stimulation leads to chronic active BCR signaling that is characterized by immobile clusters of the BCR on the cell surface and the activation of downstream signaling pathways such as NF-κB. In contrast, tonic BCR signaling seems to be antigen independent and required for the survival of mature B-cells and leads to activation of the phosphatidylinositol 3-kinase (PI3K) pathway [27,31,32,33]. Downstream mutations affecting the scaffolding protein CARD11 occur in roughly 10% of ABC DLBCLs and are associated with constitutive assembly of the CARD11-BCL10-MALT1 (CBM) signaling complex, leading to constitutive canonical NF-κB activation [26]. An alternative mechanism to activate the NF-κB signaling cascade is gain-of-function *MYD88* mutations that are detectable in almost 40% of primary ABC DLBCL samples [30]. At last, different groups reported inactivation of the ubiquitin-modifying protein A20 that acts as a negative regulator of NF-κB signaling [28,29].

The addiction to constitutive NF-κB signaling is not only important for the understanding of the molecular pathogenesis of ABC DLBCL, but also might have significant clinical implications (Figure 1). Inhibition of NF-κB using a dominant active form of IκBα or a dominant negative form of IKKβ is toxic to ABC DLBCL cell line models [20]. In the same lines, a small molecule inhibitor of IKKβ is toxic to ABC DLBCL cell lines, confirming that ABC DLBCLs are dependent on oncogenic NF-κB signaling [34]. However, inhibition of downstream NF-κB might be too toxic, and thus other proteins that act upstream of the NF-κB family members might be better suitable as molecular targets.

A large-scale RNA interference screen indicated the crucial role of CARD11 and MALT1 for survival of ABC DLBCL, as downregulation of these molecules induced toxicity in a variety of ABC DLBCL cell line models [35]. However, it is very challenging to target the scaffolding protein CARD11 pharmacologically. Thus, it is currently unclear, if the addiction to CARD11 signaling can be utilized therapeutically. In contrast, it has been shown that the paracaspase MALT1 that also acts as an Arg-specific protease, might potentially represent a promising target for the treatment of ABC DLBCL patients [35,36,37,38,39,40]. MALT1 promotes downstream NF-κB activation by both its scaffolding function and its proteolytic function by which negative NF-κB regulators are cleaved and inactivated [41]. Specifically, MALT1 cleaves among others A20, CYLD, as well as the NF-κB subunit RelB that can act as an inhibitor of NF-κB signaling [42,43,44]. Especially the introduction of novel small molecule inhibitors of MALT1 yielded promising *in vitro* and *in vivo* results in ABC DLBCL models [38,39]. Blocking the MALT1 protease function resulted in inhibition of NF-κB signaling and moreover induced toxicity in ABC DLBCL cell lines and xenograft mouse models [38,39]. Thus, testing the efficacy of these inhibitors in affected patients in early clinical trails might be warranted.

The BCR and its downstream signaling cascade seem to be an attractive molecular target for ABC DLBCL patients. BCR signaling leads not only to activation of NF-κB signaling, but potentially also to activation of other oncogenic pathways such as the PI3K/mTOR pathway, the mitogen-activated protein kinase (MAPK) pathway as well as the Nuclear factor of activated T-cells (NFAT) pathway (Figure 1). Interestingly, previous worked showed that the PI3K and the NF-κB signaling pathway functionally interact, as pharmacologic inhibition of PI3K significantly reduced NF-κB activity in ABC DLBCL cell lines [45]. A variety of different specific inhibitors can be utilized to inhibit these different signaling cascades (Figure 1). Recent preclinical and clinical data suggested that inhibition of chronic BCR signaling might be a promising therapeutic strategy. The protein kinase C (PKC) inhibitor sotrastaurin induced cell cycle arrest and/or cell death in ABC DLBCL cell lines and in a mouse xenograft model [46]. Similarly, the specific Bruton’s tyrosine kinase (BTK) inhibitor ibrutinib was toxic to preclinical models of ABC DLBCL. In contrast GCB DLBCLs were not affected by treatment with ibrutinib [27]. These data were confirmed clinically in a recent multicenter phase-II study investigating the efficacy of ibrutinib as single agent. More than 40% of relapsed and refractory ABC DLBCL patients responded to this therapy, whereas only 5% of GCB DLBCL patient showed an objective response to ibrutinib [47]. However, despite these encouraging results, ibrutinib does not seem to be curative in general in these patients, as relapses occur frequently. Thus, combinations of different pathway inhibitors might potentially overcome these relapses. A recent high-throughput screen identified various compounds such as PI3K or BCL2 inhibitors that can be combined favorably with ibrutinib [48].

Mutations affecting *MYD88* that encodes an adaptor protein have been shown to activate downstream NF-κB signaling [30]. Consequently, ABC DLBCL models depend in their survival on the expression of MYD88. Roughly 30% of all ABC DLBCLs harbor the MYD88 L265P mutation in the TIR domain. The MYD88 L265P mutation leads to spontaneous assembling of a protein-signaling complex consisting of MYD88, IRAK1, and IRAK4, leading to phosphorylation of IRAK1, NF-κB activation, STAT3 activation, and secretion of Il-6, IL-10, and interferon-β [30]. Intriguingly, a small-molecular inhibitor of IRAK1 and IRAK4 kinase activity was toxic specifically to ABC DLBCL models [30].

While the genetic abnormalities causing aberrant NF-κB signaling in ABC DLBCL are relatively well studied, the knowledge on the contribution of specific NF-κB family members to the control of the NF-κB gene expression network is limited. Recently, it has been shown that expression of the atypical nuclear IκB protein IκB-ζ (MAIL) is essential for survival of ABC DLBCL models as well as for nuclear NF-κB activity [49]. The expression of IκB-ζ itself is induced by NF-κB. Gene expression profiling experiments showed that IκB-ζ controls a large number of known NF-κB target genes most likely by interacting with the NF-κB subunits p50 and p52, respectively [49]. Intriguingly, IκB-ζ was not expressed in other NF-κB dependent hematologic malignancies such as Hodgkin’s lymphoma or multiple myeloma, indicating that IκB-ζ plays a selective role in mediating and controlling NF-κB activity in ABC DLBCL [49].

A subset of primary ABC DLBCL patient samples is characterized by high STAT3 expression [50,51]. This STAT3-high ABC DLBCL subtype had higher NF-κB activity as well as higher proliferation and glycolosis [50]. Interestingly, pharmacologic or RNA interference mediated STAT3-inhibition induced toxicity in ABC DLBCL cell lines and xenograft mouse models [50,51,52] suggesting that inhibition of STAT3 might be a promising therapeutic approach in ABC DLBCL.

Another characteristic event in the biology of ABC DLBCLs, which is potentially associated with therapy resistance, is the deregulation of different anti-apoptotic members of the BCL2 family. BCL2 itself is frequently amplified and overexpressed in ABC DLBCL models and primary patient samples [17,53]. BCL2 overexpression can potentially be utilized therapeutically by using specific BCL2 inhibitors. ABT-199 showed promising activity in different preclinical models. Interestingly, high BCL2 expression predicted sensitivity to ABT-199 suggesting that this compound should be tested in clinical trials in the setting of ABC DLBCL [54]. Another member of the BCL2 family, MCL1 is also frequently deregulated in ABC DLBCL. Roughly 25% of primary ABC DLBCLs harbor *MCL1* locus gains or amplifications. These gains/amplifications are associated with MCL1 overexpression [55]. Alternatively, MCL1 can be upregulated by constitutive STAT3 signaling in ABC DLBCL [55]. Functional data indicated that MCL1 expression is associated with resistance to conventional chemotherapeutic agents such as doxorubicin, etoposide or vincristine that are commonly used in DLBCL therapy. MCL1 knockdown using RNA interference was toxic to ABC DLBCL cell lines. Chemotherapy resistance in ABC DLBCL might potentially be overcome by pharmacologic inhibition of MCL1, as treatment with the BH3-mimetic obatoclax induced apoptosis in MCL1-positive ABC DLBCL models [55]. In summary, these data suggest that targeting anti-apoptotic BCL2 family members might represent a promising strategy in subsets of ABC DLBCL patients.

Collectively, ABC DLBCLs are characterized by a variety of different genetic aberrations. It is conceivable that these alterations contribute to the poor outcome of affected patients. Thus, targeting these abnormalities using specific small molecule compounds might improve prognosis of ABC DLBCL patients.

## 3. Potential Targets for the Treatment of GCB DLBCL Patients

GCB DLBCLs are derived from germinal center B-cells and they accordingly express germinal center B-cell genes such as *BCL6* or *LMO2* [3,9]. BCL6-positive DLBCLs are effectively killed by a small-molecule inhibitor of BCL6, suggesting that BCL6 inhibition might be successfully utilized in BCL6-positive DLBCLs [56]. GCB DLBCLs are additionally frequently characterized by addiction to the PI3K/AKT pathway that is caused by either loss of the tumor suppressor PTEN or by BCR signaling [57,58]. Thus, PI3K inhibitors might be efficacious in GCB DLBCL patients. Other novel therapeutic strategies might be inhibition of BCL2, as roughly 45% or GCB DLBCLs harbor *BCL2* translocations as well as inhibition of EZH2 [9,59]. Gain-of-function *EZH2* mutations are detectable in roughly 22% of GCB DLBCL patient samples and specific EZH2 inhibitors showed to be active in preclinical models of DLBCL [59,60,61].

## 4. Conclusions

The understanding of the driving molecular events of ABC DLBCL lymphomagenesis has significantly improved in the last couple of years. Various novel mutations have been identified that lead to constitutive activation of different oncogenic signaling pathways. Numerous clinical trials are currently being performed that investigate the efficacy of novel specific compounds. However, to understand responses observed in these studies, it is mandatory to incorporate scientific analyses including gene expression profiling. In the past, especially the assignment of patient samples into cell of origin subgroups was difficult due to the necessity of fresh frozen tissue. However, recently significant progress has been made to use formalin fixed paraffin embedded material to correctly diagnose the molecular subtype. Due to the introduction of novel techniques such as the NanoString technology, the classification into ABC and GCB DLBCL most likely will become standard clinical care [62,63].

Various studies have shown that cell of origin classification independent features of DLBCL are of scientific and clinical importance. Monti and colleagues identified three DLBCL subgroups termed “oxidative phosphorylation”, “B-cell receptor/proliferation”, and “host response” that are characterized by the virtue of specific gene expression profiles [8]. Furthermore, it has also recently been shown that copy number alterations that alter the p53 pathway are associated with inferior survival [64]. It will be challenging to integrate these different approaches. However, the combination of these datasets might eventually lead to the identification of clearly defined subgroups of patients that benefit from targeted agents leading to more specific and less toxic treatment regimens.
